# Understanding how virtual care has shifted primary care interactions and patient experience: A qualitative analysis

**DOI:** 10.1177/1357633X231167905

**Published:** 2023-04-18

**Authors:** Kelly Wu, Marlena Dang Nguyen, Geneviève Rouleau, Rhea Azavedo, Diya Srinivasan, Laura Desveaux

**Affiliations:** 1 Women's College Hospital Institute for Health Systems Solutions and Virtual Care, Toronto, Ontario, Canada; 2 Institute for Better Health, 5543 Trillium Health Partners, Mississauga, Ontario, Canada; 3 Institute for Health Policy, Management & Evaluation, University of Toronto, Toronto, Ontario, Canada

**Keywords:** Telehealth, telecare, primary care, patient experience, virtual care

## Abstract

**Introduction:**

The widespread and rapid implementation of virtual care has introduced evolutionary changes in the context, process, and way primary care is delivered. The objectives of this study were to: (1) understand whether and how virtual care has shifted the therapeutic relationship; (2) describe the core components of compassionate care from the patient perspective and (3) identify how and in what circumstances compassionate care might be amplified.

**Methods:**

Participants living in Ontario, Canada were eligible if they had interacted with their primary care clinician following the rapid implementation of virtual care in March 2020, irrespective of virtual care use. One-on-one semi-structured interviews were conducted with all participants and data were analyzed using inductive thematic analysis.

**Results:**

Four themes emerged across 36 interviews: (1) Virtual care shifts communication patterns but the impact on the therapeutic relationship is unclear; (2) Rapid implementation of virtual care limited perceived quality and access among those who did not have the option to utilize it; (3) Patients perceive five key elements as central to compassion in a virtual context; and (4) Leveraging technology to fill gaps within and beyond the visit is a step towards improving experiences for all.

**Discussion:**

Virtual care has transformed the ways in which patient-clinician communication operates in primary care. Patients with access to virtual care described largely positive experiences, while those whose interactions were limited to phone visits experienced decreased quality and access to care. Attention must shift to identifying effective strategies to support the health workforce in building virtual compassion competencies.

## Introduction

The primary care landscape changed dramatically with the rapid uptake of virtual care in response to the coronavirus disease 2019 (COVID-19) pandemic. Ontario, Canada recorded a significant increase of video visits from 1.2% of all primary care visits (i.e., office, home, and video visits) in March to July 2019, to 71.2% constituting virtual visits during the same months in 2020.^1^ The widespread and rapid implementation of virtual care has introduced evolutionary changes in the context, process and way health care is delivered,^2^ much of which are still being explored.

Virtual care involves any remote interaction between patient(s) and members of their circle of care that is facilitated by technology (i.e., video conferencing, emailing, instant messaging).^3^ While it is increasingly leveraged to provide a greater number and variety of modalities for communication between patients and clinicians, there are concerns around the impact of virtual care on the therapeutic relationship.^4^ The push towards efficiency and accessibility has overlooked the importance of establishing a human connection,^5^ which is central to establishing the bond that between patients and clinicians – a bond that is often described as the hallmark of primary care.^6,7^ When implemented well, technology can create efficiencies that encourage clinicians to focus more time on their relationship with the patient in front of them.^8^ This is essential to the ability to both recognize and respond to suffering, which is fundamental to providing compassionate care.^9^ While virtual care has been central to healthcare delivery in Ontario for almost three years, its impact on the relationships and the experience of compassion remains unclear.

As the use of virtual care becomes further normalized, attention must be paid to gaps in our understanding of how connectivity is established and how compassion is experienced without losing sight of the digital divide.^10^ Therefore, the objectives of this study were to: (1) understand whether and how virtual care has shifted the therapeutic relationship; (2) describe the core components of compassionate care from the patient perspective and (3) identify how and in what circumstances compassionate care might be amplified for both those who use virtual care and those who do not. This study focused on primary care practices due to the longitudinal, relationship-based nature of care.

## Methods

This qualitative study involved one-on-one, semi-structured interviews with patients who had interacted with their primary care clinician (i.e., family doctors, registered nurses and nurse practitioners), following the rapid implementation of virtual care. Interview questions aimed to explore how technology influences patients’ interactions with their primary care clinician with a focus on understanding how virtual care can amplify compassionate care for patients (see Supplementary file 1). This study was formally reviewed by institutional authorities at Women's College Hospital (WCH) and was deemed not to require Research Ethics Board approval under the Assessment Process for Quality Improvement Projects pathway (APQIP # 2021-0028-P). The reporting of this study was guided by the Consolidated Criteria for Reporting Qualitative Research Checklist.^11^

### Study setting

Primary care clinicians are considered the first point of contact for patients to the healthcare system, delivering relationship-based and longitudinal care across the lifespan.^12^ Ontario has a publicly funded, single-payer health care system for permanent residents to access most medically necessary services covered under the Ontario Health Insurance Plan, which includes access to primary care.^13^ Patients can receive virtual care from their primary care clinicians in various forms including video calls, asynchronous messaging and remote monitoring for some conditions.^14^

### Sampling and participant recruitment

Participants were eligible if they lived in Ontario, Canada. We recruited two patient groups – those with *any* virtual care experience with their primary care clinician and those with no experience using virtual care (i.e., no experience using technology in a healthcare context). A multi-method sampling strategy was used, including purposive sampling, snowball sampling, and passive recruitment materials. As the study sought to speak to diverse patient groups, the recruitment strategy was further refined through consultation with Equity-Mobilizing Partnerships in Community (EMPaCT), a patient-partnership model that centers the voices and perspectives of diverse patients and their lived experiences housed at WCH and supported by the WCH Institute for Health System Solutions and Virtual Care (WIHV). EMPaCT provided a health equity assessment for the study protocol and advised on best-practice recruitment strategies for ways to reach diverse communities. The research team refined participant-facing materials and recruitment activities in line with EMPACT recommendations, such as removing jargon in the interview guide and reaching out to specific community organizations (see Supplementary file 2) for study advertisement.

Study information was shared on social media platforms (i.e., Twitter, LinkedIn and Facebook) and recruitment materials directed interested participants to reach out to the primary study contact by email to determine eligibility for the study. EMPaCT members facilitated connections with key communities of interest, with the project team leading recruitment activities in these communities in line with EMPaCT recommendations. These included reaching out to Patient Advisory Groups, Family Health Teams, Ontario Health Teams as well as other specific community organizations (see Supplementary file 3 for full list). A total of 14 community organizations were sent recruitment materials (i.e., promotional posters and recruitment scripts) and 3 responded indicating a willingness to distribute. All recruitment activities were led by the research team using multiple strategies in parallel, including email, social media platforms and community-driven strategies identified by patient engagement groups. Eligible participants who contacted the study team were sent a Study Information Letter and Consent Form by email at the time of scheduling the interview, and verbal consent was obtained prior to the start of the interviews. A follow-up recruitment email was sent within 2 weeks of the original invitation. Researchers had no established relationship with any participants before the start of the study.

While the aim of the study was to hear from diverse patients, resource constraints limited the study recruitment and data collection to participants who spoke English.

### Data collection

Individual qualitative semi-structured interviews with patients were conducted by one member of the research team (KW, MDN) between April and November 2021. Interviews were conducted over an online video-conferencing platform (Zoom). A second team member (RA) was present with their camera and microphone turned off to take notes and provide an additional perspective beyond the interviewer to support data collection and analysis. Recruitment continued until thematic saturation was determined to be reached, meaning little to no new comments or insights emerged during the interviews that either refined or challenged existing insights or categories of insights within the data sample.^15^

Interview guides were tailored to each participant group for data collection. Interviews with patients who had experience with virtual care included questions to understand their experiences with compassionate care, their perceived challenges and benefits of virtual care, and perspectives on how to best utilize virtual care with respect to compassionate care. Interviews with patients who had no experience with virtual care also explored their perspectives on compassionate care and virtual care. The interview guide was reviewed with EMPaCT and revised based on feedback but was not individually pilot tested with patients. All interviews were audio-recorded, anonymized and transcribed verbatim by a third-party. The transcripts were not returned to participants for comment.

Participants were informed that participation in the interview was completely voluntary, and they could withdraw from the study at any time without penalty. Verbal consent was obtained before the start of interviews and all participants received an electronic gift-card (CAD $30) as a token of appreciation. Demographic information, including age, gender, ethnicity, and geographic location was collected prior to the interview.

### Data analysis

Qualitative data were analyzed using an inductive thematic analysis approach using a six-phased method that was conducted iteratively.^16,17^ In phase 1, the research team was deeply engaged with the data from reading the transcripts to documenting any emerging thoughts and reflections. For phase 2, members of the research team (KW, MDN, RA) generated initial codes and then developed a preliminary coding framework for each participant group by coding a subset of 3 transcripts per group. NVivo 12 software (QSR International) was used to assist with coding and analysis. The remaining transcripts were single coded by two research team members (KW, MDN) and the team met regularly to iteratively refine the codebook to reflect new codes, merge related codes, and resolve discrepancies. In phases 3-5, codes were regrouped together into preliminary themes independently for each participant group (patients who have engaged with and those who have not) and were mapped back to the study objectives. Findings were then synthesized to find areas of similarities and differences. Miro, an online whiteboard collaboration tool, was used to aid the research team in visually representing the preliminary themes, identifying relationships between themes, and as a consultation strategy to gather feedback and reflections on findings. Refinements and specifications of themes and their relationships were conducted for each of the two participant groups, after which the themes from both groups were compared for areas of convergence and divergence to identify additional insights in line with study objectives. Members of the research team (LD, KW) prepared thematic summaries for each participant group to support iterative refinement of themes and supported by key quotes. A total of 2 thematic summaries were produced. An audit trail was kept of all team meetings using audio recordings and meeting minutes and storing all versions of the coding framework and thematic summaries.

## Results

A total of 36 interviews were conducted, including 23 patients who engaged with virtual care and 13 patients who had not, of which 13 provided feedback via member-checking. The interviews ranged from 25 min to 71 min in duration (average = 44 min). Participant characteristics can be found in Table 1. The analysis identified key aspects of patient experience and the contextual factors that influence and inform participant perspectives about virtual care and its connection with compassionate care.

**Table 1.** Participant demographics.

	Patient *(N = 36)*
Age n(%)	
* 18–20*	4 (11)
* 21–29*	8 (22)
* 30–39*	10 (28)
* 40–49*	5 (14)
* 50–59*	1 (2.8)
* 60+*	5 (14)
* Unidentified*	3 (8.3)
Gender n(%)	
* Female*	23 (64)
* Male*	13 (36)
Ethnicity n(%)	
* Black – African*	3 (8.3)
* Black – Caribbean*	1 (2.8)
* Black – North American*	5 (14)
* East Asian*	2 (5.6)
* White European*	17 (47)
* Latin American*	1 (2.8)
* Middle Eastern*	0 (0)
* Mixed heritage*	0 (0)
* South Asian*	6 (17)
* Unidentified*	1 (2.8)
Geography n(%)	
* Central East Ontario*	24 (67)
* Central West Ontario*	3 (8.3)
* Eastern Ontario*	5 (14)
* Northern Ontario*	0 (0)
* Western Ontario*	3 (8.3)
* Unidentified*	1 (2.8)

### Qualitative themes

#### Theme 1: virtual care shifts communication patterns but the impact on the therapeutic relationship is unclear

*Patients who engaged with virtual care* reported changes in how they related to and communicated with their clinician, however most reported it was too early to identify whether things had shifted. Changes in communication included discernably shorter and rushed conversations compared to their in-person experiences, with less small talk and the impression of being limited to one health concern per visit. In contrast, some participants felt the virtual interactions provided more space to prepare and a more focused, uninterrupted conversation with their clinician that fostered a sense of being responded and attended to.

“*I think just recognizing that we kind of have to again read between the lines a little bit more and kind of parse out these feelings that I’m having a little bit more because it's going to take me longer to be able to express myself. But of course, on a digital platform, people think the opposite, that it can be a quicker visit because we’re getting to the point so much faster. So I think spending a little bit more time creating those relationships is [important].” P2, virtual experience*

“*Another thing that I kind of had, sometimes I would be nervous when I would see the doctor, like in person, and I would forget to ask certain things. But this time, because I was on my laptop, like I kind of had my questions ready to go and I had them on the side. So I was just looking at the screen and I was just asking him more questions based off of that. Whereas, in person I would be a little bit nervous to take out my phone and go through it and be like OK, I have all these questions.” P10, virtual experience*

Patients with a previously established therapeutic relationship felt more supported and at ease when transitioning to virtual care and attributed this to the familiarity with their clinician. This created confidence that virtual care would augment the quality of their care rather than undermine it. In contrast, patients who met their clinician for the first time in a virtual interaction felt less confident in approaching communication via a virtual platform.

“*I feel like I need to connect with someone, you’re taking care of me, this has to do with my life, it has to do with my health, it's a priority for me. So, me placing myself in your hands and asking for your opinion or for your services with regards to my health, is like me putting my life with you. So, I feel like I have to have a particular connection with that person or - I just don't know. I just feel like I've got to be close with that person, or it's actually someone I trust - I don't know how to really explain this. It's probably because I'm used to having him for a long while, so I'm not used to new faces or new people taking care of my treatments and all that.” T5, no virtual experience*

“*There was a huge almost mistrust in that relationship just because I didn’t know them and didn’t feel comfortable with them seeing my background and talking about really personal things having never met them in person.” P2, virtual experience*

#### Theme 2: rapid implementation of virtual care negatively impacted perceived quality and access among those who did not have the option to utilize it

Patients who had not engaged in virtual care reported a noticeable shift in the patient-clinician relationship, with most describing that opportunities to interact with their clinician were limited to telephone calls. This group expressed an openness and interest in virtual care and described several perceived benefits, including saving time, not having to wait in a waiting room, and a reduced commute. While there was diversity in cultural background and lived experiences across this group of participants, there was alignment on the perception that virtual care could help support urgent care needs and increase availability broadly.

“*But I would be interested if my computer here was able to take the information and do it. I have an old computer, I’m not that updated with everything, but I would be open to using it and having video conferences like this with a doctor, if that's what you’re meaning, or a health specialist. I would be totally open to that because it saves me time going and sitting in a waiting room and it save them time waiting to come and go from the office and miss appointments and all that type of thing.” T2, no virtual experience*

These participants acknowledged the importance of tailoring virtual care to patient beliefs, experiences, and preferences to ensure equitable access. For example, patients living in multigenerational households described the value placed on in-home visits to see multiple family members at once or having a clinician who practices in a family health team to access additional supports and resources (e.g., after-hours care, community paramedicine). The shift to virtual care during the pandemic had left some patients without access to this tailored approach to care, which undermined continuity and connectivity. Other patients described challenges to communicating by telephone due to language barriers and having fewer visual cues to indicate confusion or lack of understanding to their clinician.

“*I’m thinking virtually, you know if they saw us, we’d be sitting side by side and they could, maybe they could see that I’m listening and I’m looking to him and they would maybe see our relationship and they would see him look to me sometimes for an answer. I feel that when I’m answering it's like, I’m not letting him talk, but they would maybe see that he would look to me to answer a question rather than me jump in.” T1, no virtual experience*

Finally, some patients explained that they would prefer visits with their regular clinician with whom they have a strong relationship where care was naturally tailored over more transactional interactions with an unfamiliar clinician on virtual care.

“*When you've been getting treatment for a particular person for years, for a long while and then someone – you not able to reach the person or the person is not available at that particular moment, then you just see a particular stranger to treat you and all that. Although the person you're also seeing is certified and all that, but then it's not the same because you're already familiar with this person, you're used to this person treating you and all that. So it's like a new pain, it's like a new experience. And it's not easily advisable changing health care, changing doctors in that case.” T5, no virtual experience*

#### Theme 3: patients perceive five key elements as central to compassion in a virtual context

Patients noted that connection and trust were the foundation of their confidence in clinicians and clinician recommendations. Patients further highlighted five key elements of compassionate care: patient autonomy, holistic care, patient security, encounter-based presence, and a collaborative approach (see Table 2). Underlying these elements were patient beliefs that it is the clinician's role to provide the patient with options, engage them as an equal in conversations, and coordinate with other health professionals to arrange additional testing and referrals to ensure proper continuity of care. They expected that clinicians have an in-depth understanding about the technology being used during digital health interactions.

**Table 2.** Patient-identified elements of compassionate care.

Element	Description	Supporting quote
Autonomy	The availability of choice and ability to make an informed, independent decision.	*“And this is something that could be engaged by healthcare clinicians. “Under what circumstances would you be comfortable? Teleconferencing? Videoconferencing? And seeing me in person?” It's part of the education process so that patients themselves realize, “Yeah. That's true, when this was happening, this was important to me.” So that they themselves learn about their needs and convey that to healthcare clinicians. We need to have that dialogue moving forward.” P21, F, virtual experience*
Holistic care	Care that extends beyond the presenting symptom(s) to include the patient's context and broader needs.	*“They are a person who is also learning from you about what's important, what your values are, what issues are most concerning to you, what your resources are. And if there's trust, you’re more apt I think to be open about all of those areas that will impact your health. So, I think being humanistic means connecting on a personal level, building trust and ensuring that both partners in this healthcare relationship can contribute to positive outcomes.” P25, virtual experience*
Security	Feeling physically and psychologically safe.	*“Another thing that I kind of had, sometimes I would be nervous when I would see the doctor, like in person, and I would forget to ask certain things. But this time, because I was on my laptop, like I kind of had my questions ready to go and I had them on the side. So I was just looking at the screen and I was just asking him more questions based off of that. Whereas, in person I would be a little bit nervous to take out my phone and go through it and be like OK, I have all these questions.” P10, virtual experience*
Encounter-based presence	Feeling prioritized by and connected to the clinician during the visit.	*“I feel like I need to connect with someone, you’re taking care of me, this has to do with my life, it has to do with my health, it's a priority for me. So me placing myself in your hands and asking for your opinion or for your services with regards to my health, is like me putting my life with you. So I feel like I have to have a particular connection with that person or - I just don't know. I just feel like I've got to be close with that person, or it's actually someone I trust out - I don't know how to really explain this. It's probably because I'm used to having him for a long while, so I'm not used to new faces or new people taking care of my treatments and all that.” T5, no virtual experience*
Collaborative approach	Being engaged as an equal in the conversation, including mutual exchanges of knowledge.	*“OK asides my family doctor I believe that whatever relationship person or individual has with any health care clinician should be a partnership. Every person should have a say in his or her health. So it should definitely be a partnership. It shouldn’t be one within the, the physician shouldn’t be telling you everything you must do. I believe even in the medical space there are options for therapies and then you know consider the options as a patient and see which best fits your needs.” P18, no virtual experience*

Unfortunately, these expectations were not realized during the early implementation of virtual care. Patients reported they were not given a choice regarding virtual modalities (i.e., phone versus video) and most described poor experiences with telephone consults due to increased reliance on patient report and explanation (in the absence of visuals), a lack of relational connection, and invisibility of their caregivers. All patient participants expressed that providing choice of modality would give them more agency in their care. While telephone was often presented as the more accessible option, several participants described the negative but avoidable experience introduced by immature technology or inconsistent workflows.

“*One unfortunate thing that happened that really made me mad was we were having an appointment by telephone for my husband and first we talked to the fellow and that was fine. And then the routine was the fellow will hang up and then the doctor will call back. So we’re both sitting in the living room waiting for this call back and the call dropped. And they didn’t try again and they wrote in his report, in the notes, we tried to call Mr [name] and he did not answer the phone. Like are you kidding me? It makes it look like we didn’t care about the appointment. I was really insulted about. You’re going through a really hard time and you read that in your file. Like we didn’t care to follow up. I mean this is the person's life right? It's really important.” P26, virtual experience*

#### Theme 4: leveraging technology to fill gaps within and beyond the visit is a step towards improving experiences for all

Participants highlighted several opportunities to leverage technology both within and beyond the boundaries of a virtual interaction. Within the visit, leveraging technology included using video to establish direct eye-contact and demonstrate attentiveness. It also enables patients to leverage a visual to support communicating symptoms that would otherwise be difficult to express or describe. One participant described the unanticipated value of being face-to-face virtually compared to in-person visits:

“*One thing that I also found interesting was, when I would always be in his office, he wouldn’t really be facing me because he was constantly typing at the computer. But this time, because it was virtually, like I didn’t really feel that because, you know, he's sitting in front of a computer screen regardless, and this time his back wasn’t kind of turned away from me. So, it felt a little bit better to be quite honest, kind of having somebody interacting face to face with you.” P10, virtual experience*

It was broadly acknowledged that in instances where virtual care has promoted positive patient experience, it has required increased clinician effort and investments in new workflows. Participants described that creating workflows that enable a seamless experience without interruptions, including technology navigation support for patients, is important for patients to feel comfortable discussing sensitive topics.

“*I know that's out of their control, but it freezes and then I’m sitting there frustrated because I’ve worked up the courage to discuss my issues and now all of a sudden how anxiety-provoking is it that their screen is frozen and I don’t know if the call is going to end, I don’t know if they’re going to call me on the phone. There's not really a solid backup plan in place to navigate those issues.” P2, virtual experience*

Leveraging technology beyond the visit was perceived to build upon a compassionate interaction to create a compassionate experience within the healthcare system. For example, operationalizing (and responding to) asynchronous messages signals availability, checking-in about a previous concern signals both commitment and attentiveness, and sending a prescription or imaging requisition directly to the patient signals respect for the patient's time. Additional suggestions included online booking and displaying accurate wait-times.

## Discussion

This study describes how virtual care has transformed the ways in which patients and their primary care clinicians communicate with each other and how this relates to the experience of compassionate care. Although pre-existing therapeutic relationships supported positive experiences among those who accessed virtual care, patients who did not have the option to engage in virtual care experienced decreased quality and access to care irrespective of existing relationships. Our data emphasizes the broad support for patient-centered virtual care and the expansive opportunities virtual care presents to enable for compassionate primary care.

As communication in healthcare continues to evolve, virtual care has the potential to diversify the number of pathways through which patients and clinicians interact. Patient expectations continue to evolve as capabilities for bidirectional communication are increasingly available through a range of mechanisms including video visits and asynchronous messaging.^18^ Increased feelings of psychological safety in virtual interactions leads patients to experience these interactions as more honest and empathetic compared to in-person consults.^19^ The ability to connect is highly reliant on non-verbal cues,^20^ and listening and paying attention are central to how patients experience compassion,^21^ emphasizing the need to build competency in compassionate verbal and non-verbal communication behaviours in a virtual context.^22^ Key behaviours include avoiding jargon, making eye contact, asking open-ended questions, setting expectations, engaging in small talk, including caregivers, following up on prior concerns, asking probing questions, and acknowledging facial expressions and body language.^23^

As our understanding of how patients experience compassion is advancing,^21,23^ we must shift to identifying effective strategies that support the development of virtual compassion competencies among clinicians and the related operational capacity needed to ensure the health workforce is able to convey compassion during virtual care delivery. Online training and coaching and virtual reality training are promising approaches to building clinical competency through virtual formats,^24–26^ thereby building skills in the context in which they are utilized. Virtual training can positively impact comfort and preparedness for virtual interactions by addressing technical proficiency, examination skills, and communication, however, approaches for developing nonverbal communication skills requires further attention.^27^ Given the possibility for misalignment between clinician and patient experience,^28^ evaluations of training modules should include fidelity of receipt (the patient experience of self-reported improved competence) at the patient level.

The COVID-19 pandemic disproportionately impacted populations who have been historically marginalized, including low-income and racialized minority groups,^29^ and the parallel implementation of virtual care was no exception. To realize equitable virtual care implementation, system level strategies must simplify complex interfaces and workflows using supportive intermediaries,^30^ as well as creating mechanisms for patient engagement in the planning and delivery of virtual care.^30,31^ Policy priorities include identifying clear health system leadership accountable for embedding virtual care across the care continuum and leveraging incentives such as outcome-based payment models to achieve health system improvements.^3^ Practice guidelines should include specificity around how virtual care can be tailored to structurally marginalized groups, including reducing stigma, bias, and discrimination to encourage access and providing more opportunity for culturally inclusive practices (e.g., Indigenous patients may smudge in their own environments before an appointment).^32^ Less than 50% of households in rural areas meet the basic universal service target required by the Canadian Radio-television and Telecommunication Commission.^33^ Policies to close the gap on structural inequities include ensuring access to the required infrastructure (i.e., broadband internet access, speed, and costs) to enable participation in virtual care are critical for those residing in southwestern Ontario, northern and Indigenous communities.^34^ Future research and system efforts should focus on identifying the infrastructure investments needed to streamline and simplify the use of video consultations.^20,22^

Participation in the qualitative interviews was voluntary which introduces the potential of selection bias. To mitigate this, we consulted EMPaCT and reached out to several Patient Advisory Groups to facilitate purposive sampling as well as including participants that had no engagement with virtual care. Given the restrictions of the COVID-19 pandemic at the time of this study, we were not able to have a physical recruitment presence in the community and therefore recruitment occurred solely via social media and digital distribution which may introduce bias into our sample. The findings of this study serve as a foundation for future research aimed at achieving a broader understanding of how virtual care works for different patients across a range of social and cultural contexts. Our findings are not generalizable to individuals who do not speak English or have access to an English-speaking caregiver, emphasizing the need to build on this work by purposively engaging non-English speaking individuals, those without access to technology (who were unlikely to hear about our study), and those from Indigenous and Black communities. We were unable to evaluate the impact of virtual care on the therapeutic relationship due to the relatively infrequent engagement participants had with their primary care clinician between the rapid uptake of virtual care (March 2020) and study recruitment (April-November 2021). Future research should focus on whether the identified shifts in communication were maintained and, if so, their impact on the therapeutic relationship from the perception of patients and clinicians.

## Conclusion

Despite the connectivity challenges introduced by the COVID-19 pandemic, the widespread introduction of virtual care represents a positive shift in healthcare delivery. The implementation of virtual care and associated workflows within primary care outpaced the ability to understand the skills needed to provide high quality, compassionate experiences in this context. Patients clearly described five key elements of compassionate virtual care, which center around their experience of both the care provided and their relationship with the clinician providing care. As health systems move towards routinized use of virtual care, there is a need to understand where and how virtual care creates value and for whom. Additionally, future work should identify and co-design strategies to (1) support clinicians in building compassion-oriented virtual care competencies and (2) leverage technology within and beyond the boundaries of a visit to improve experiences for all patients.

## Supplemental Material

sj-docx-1-jtt-10.1177_1357633X231167905 - Supplemental material for Understanding how virtual care has shifted primary care interactions and patient experience: A qualitative analysis Supplemental material, sj-docx-1-jtt-10.1177_1357633X231167905 for Understanding how virtual care has shifted primary care interactions and patient experience: A qualitative analysis by Kelly Wu, Marlena Dang Nguyen, Geneviève Rouleau, Rhea Azavedo, Diya Srinivasan, and Laura Desveaux in Journal of Telemedicine and Telecare

## References

[1] Glazier RH Green ME Wu FC , et al. Shifts in office and virtual primary care during the early COVID-19 pandemic in Ontario, Canada. CMAJ 2021; 193: E200–E210. 10.1503/cmaj.202303 PMC7954541 33558406

[2] Wiljer D Charow R Costin H , et al. Defining compassion in the digital health age: Protocol for a scoping review. BMJ Open 2019; 9: e026338. 10.1136/bmjopen-2018-026338 PMC6398782 30772865

[3] Shaw J Jamieson T Agarwal P , et al. Virtual care policy recommendations for patient-centred primary care: Findings of a consensus policy dialogue using a nominal group technique. J Telemed Telecare 2018; 24: 608–615. 28945161 10.1177/1357633X17730444

[4] Breton M Sullivan EE Deville-Stoetzel N , et al. Telehealth challenges during COVID-19 as reported by primary healthcare physicians in Quebec and Massachusetts. BMC Fam Pract 2021; 22: 192. 34563113 10.1186/s12875-021-01543-4 PMC8467009

[5] Zulman DM Verghese A . Virtual care, telemedicine visits, and real connection in the era of COVID-19: Unforeseen opportunity in the face of adversity. JAMA 2021 Feb 2; 325: 437. 33528520 10.1001/jama.2020.27304

[6] Hardcastle L Ogbogu U . Virtual care: Enhancing access or harming care? Healthc Manage Forum 2020; 33: 288–292. 32686506 10.1177/0840470420938818 PMC7372098

[7] Deledda G Moretti F Rimondini M , et al. How patients want their doctor to communicate. A literature review on primary care patients’ perspective. Patient Educ Couns 2013; 90: 297–306. 22709720 10.1016/j.pec.2012.05.005

[8] Canadian Medical Association. Virtual Care in Canada: Discussion Paper. CMA Health Summit; 2019 Aug. Available from: https://www.cma.ca/sites/default/files/pdf/News/Virtual_Care_discussionpaper_v2EN.pdf

[9] Feo R Kitson A Conroy T . How fundamental aspects of nursing care are defined in the literature: A scoping review. J Clin Nurs 2018; 27: 2189–2229. 29514402 10.1111/jocn.14313

[10] Robotham D Satkunanathan S Doughty L , et al. Do we still have a digital divide in mental health? A five-year survey follow-up. J Med Internet Res 2016; 18: e309. 10.2196/jmir.6511 PMC5141335 27876684

[11] Tong A Sainsbury P Craig J . Consolidated criteria for reporting qualitative research (COREQ): A 32-item checklist for interviews and focus groups. Int J Qual Health Care 2007; 19: 349–357. 17872937 10.1093/intqhc/mzm042

[12] Haggerty JL . Continuity of care: A multidisciplinary review. Br Med J 2003; 327: 1219–1221. 14630762 10.1136/bmj.327.7425.1219 PMC274066

[13] Marchildon GP Allin S Merkur S . Health Systems in Transition Canada Health system review. 2020; 22: 2020. 33527903

[14] Canada Health Infoway. Canadian’s Health experience during Covid-19. 2020. Available from: https://www.infoway-inforoute.ca/en/component/edocman/3828-canadians-health-care-experiences-during-covid-19/view-document?Itemid=0

[15] Saunders B Sim J Kingstone T , et al. Saturation in qualitative research: Exploring its conceptualization and operationalization. Qual Quant 2018; 52: 1893–1907. 29937585 10.1007/s11135-017-0574-8 PMC5993836

[16] Braun V Clarke V . Using thematic analysis in psychology. Qual Res Psychol 2006; 3: 77–101.

[17] Nowell LS Norris JM White DE , et al. Thematic analysis: Striving to meet the trustworthiness criteria. Int J Qual Methods 2017; 16: 160940691773384.

[18] Luxton DD McCann RA Bush NE , et al. Mhealth for mental health: Integrating smartphone technology in behavioral healthcare. Professional Psychology: Research and Practice 2011; 42: 505–512.

[19] Preece J . Empathic communities: Balancing emotional and factual communication. Interact Comput 1999; 12: 63–77.

[20] De Guzman KR Snoswell CL Smith AC . Telehealth sustainability after COVID-19 – can you see me by video? Aust Health Rev 2022 Jun 17; 46: 642–643. 35710315 10.1071/AH22134

[21] Baguley SI Pavlova A Consedine NS . More than a feeling? What does compassion in healthcare ‘look like’ to patients? Health Expect 2022; 25: 1691–1702. 35661516 10.1111/hex.13512 PMC9327826

[22] De Guzman KR Snoswell C Giles CM , et al. General practitioner perceptions of telehealth services in Australia: a qualitative study. BJGP Open 2021 Nov 24. doi: 10.3399/BJGPO.2021.0182. PMC8958753 34819294

[23] Desveaux L Wu K Rouleau G , et al. Building compassionate experience through compassionate action: A qualitative behavioural analysis. JMIR Preprints 2022: 43981. 10.2196/43981 PMC10267792 37256678

[24] Kemp J Zhang T Inglis F , et al. Delivery of compassionate mental health care in a digital technology–driven age. Scoping Review. J Med Internet Res 2020; 22: e16263. 10.2196/16263 PMC7084292 32141833

[25] Rouleau G Pelletier J Côté J , , et al. Codeveloping a virtual patient simulation to foster Nurses’ relational skills consistent with motivational interviewing: A situation of antiretroviral therapy nonadherence. J Med Internet Res 2020 Jul 15; 22: e18225. 10.2196/18225 PMC7391166 32672679

[26] Formosa NJ Morrison BW Hill G , et al. Testing the efficacy of a virtual reality-based simulation in enhancing users’ knowledge, attitudes, and empathy relating to psychosis. Aust J Psychol 2018; 70: 57–65.

[27] Lawrence K Hanley K Adams J , et al. Building telemedicine capacity for trainees during the novel coronavirus outbreak: A case study and lessons learned. J GEN INTERN MED 2020; 35: 2675–2679. 32642929 10.1007/s11606-020-05979-9 PMC7343380

[28] Kennedy BM Rehman M Johnson WD , et al. Healthcare providers versus patients’ understanding of health beliefs and values. Patient Exp J 2017; 4: 29–37. 29308429 PMC5751953

[29] Price-Haywood EG Burton J Fort D , et al. Hospitalization and mortality among black patients and white patients with COVID-19. N Engl J Med 2020; 382: 2534–2543. 32459916 10.1056/NEJMsa2011686 PMC7269015

[30] Shaw J Brewer LC Veinot T . Recommendations for health equity and virtual care arising from the COVID-19 pandemic. Narrative Review. JMIR Form Res 2021; 5: e23233. 10.2196/23233 PMC8023377 33739931

[31] Nouri S Khoong EC Lyles CR , et al. Addressing equity in telemedicine for chronic disease management during the COVID-19 pandemic. NEJM Catalyst Innovations in Care Delivery 2020.

[32] Birnie K Killackey T Backlin G , et al. Ensuring equity and inclusion in virtual care best practices for diverse populations of youth with chronic pain. hcq 2022; 24: 25–30. 10.12927/hcq.2022.26778 35467507

[33] Canadian Radio-Television and Telecommunications Commission (CRTC). Communications monitoring report (2020). Available from: https://crtc.gc.ca/eng/publications/reports/policyMonitoring/2020/cmr4.htm

[34] Hambly H Rajabiun R . Rural broadband: Gaps, maps and challenges. Telemat Inform 2021 Jul; 60: 101565.

