# Supplementary material for: Understanding how virtual care has shifted primary care interactions and patient experience: A qualitative analysis
Source: J Telemed Telecare. 2023 Apr 18;31(1):73–81. doi: 10.1177/1357633X231167905 (PMC11626843; doi:10.1177/1357633X231167905)
Supplement: sj-docx-1-jtt-10.1177_1357633X231167905 - Supplemental material for Understanding how virtual care has shifted primary care interactions and patient experience: A qualitative analysis [file sj-docx-1-jtt-10.1177_1357633X231167905.docx]

**Supplementary files**

**Supplementary file 1: Interview guides**

Interview guide for patients who have engaged with virtual care

Before we begin the interview, can you please let me know whether you’ve had the chance to complete the short demographic questions sent in the online survey? (If no, will send reminder email after interview)

| **Topics** | **Questions** | **Probes** |
| --- | --- | --- |
| Introduction | Why are you interested in participating in this interview/study today? |  |
| Context setting | Before we begin the interview, we want to acknowledge how personal background and lived experiences can impact an individual’s experience with the health care system and the care they receive from their doctor or nurse. We refer to this as context. Context can be the things that patients think their healthcare providers need to be aware of. We’ll reflect on this context at the end as well but feel free to draw on these points when it makes sense for you. Please know that any topic is welcome for discussion. |  |
| Perceptions of compassionate care | For the purpose of this study, we define compassionate care as:   - Awareness of another’s experience or need - Appraising one’s own role and abilities in an interaction - Being aware of context   but we want to hear in your own words, what does compassionate care mean to you? | How do you know you’re receiving compassionate care? /what does that *feel* like to you? |
|  | To what degree has your family doctor/nurse expressed awareness of another’s (your) experience or need?    Can you walk me through what the interaction was like? | What made you feel that compassionate care was present? |
|  | To what degree has your family doctor/nurse considered their own role (responsibilities) and abilities in an interaction (with you)?  Can you walk me through what the interaction was like? | What made you feel that compassionate care was present |
|  | To what degree has your family doctor/nurse been aware of (your/their own) context (other factors/circumstances that influence your health/life)?  Can you walk me through what the interaction was like? | What made you feel that compassionate care was present |
|  | What aspects of health matter to you? |  |
|  | Primary care is unique in that you have the opportunity to interact with your family doctor/or nurse over time, how long have you known your family doctor/nurse? Can you describe the nature of your relationship with them? Do you have a strong relationship with them? | **Does** that impact your experience of compassionate care?  **Do** you think not having a strong relationship would impact your experience of compassionate care?  How did you build that relationship with your family doctor/nurse?  If relationship is not strong: What would make the relationship better? |
|  | Please complete this sentence for me:  1. As a patient my responsibility is: ________________.  2. My physicians responsibility is: _________________. | How does your perception/experiences of compassionate care align with your perception of the responsibility of your family doctor/nurse? |
| Experiences of digital care | We define digital care as any healthcare interaction that uses technology (ex. Emailing, video visits, remote monitoring, etc.). Is this definition clear? In what other ways have you received digital care with your family doctor and nurse? |  |
|  | Can you describe your experiences using technology with your family doctor/nurse? | Can you walk me through a typical virtual care visit?  What was the reason for your consultation?    What visit options were available to you and how were they presented?    What did you like, what did you not like?    How does this compare to an in-person visit? |
| Intersection of compassionate care and digital care | Within your digital care interactions, how has your family doctor or nurse shown you compassionate care?  What did your family doctor or nurse do that made you feel that it was compassionate? | Is there something they could/should have done? what was most effective? Why was it most effective? (really trying to understand on what it meant to the participant)    Has using technology influenced your experiences of receiving compassionate care? |
|  | What has your experience been like using technology to access your healthcare? | How similar is that experience with technology outside of healthcare?    Is there anything else that could be done to make you feel more comfortable with technology?    Do you have what you need in order to access care digitally? |
|  | Have you ever been asked about your experiences using technology to access care? | Has your family doctor/nurse ever asked you? |
|  | In what instances do you prefer to meet your family doctor/nurse in person versus virtually? | And how does that link back to having a compassionate interaction? |
|  | How would you describe your ideal form of virtual care? What does that look like to you? |  |
| Closing and context setting | Keeping in mind the role of context in compassionate care, how do you feel your personal background and lived experiences influences your experience of receiving compassionate care digitally? We invite you to reflect on how your race, gender and other sociodemographic factors including language, and disability may impact both the care you receive and your experiences within the healthcare system. | How does your family doctor or nurse’s background (race, linguistic, gender etc.) influence your comfort level? |
|  | Is there anything else you want to share with me about compassionate care and digital health? |  |

Those are all the questions I had. Is there anything else you would like to add on this topic that we have not already covered?

Thank you for participating.

Interview guide for patients who have not engaged with virtual care

Before we begin the interview, can you please let me know whether you’ve had the chance to complete the short demographic questions sent in the online survey? (If no, will send reminder email after interview)

1. experiences of health care 2. What does your ideal form of health care look like to you? 3. What does compassionate care mean to you? 4. is there a role for digital health to support delivering the key components of what compassionate care is to you?

| **Topics** | **Questions** | **Probes** |
| --- | --- | --- |
| Introduction | Why are you interested in participating in this interview/study today? |  |
| Context setting | Before we begin the interview, we want to acknowledge how personal background and lived experiences can impact an individual’s experience with the health care system and the care they receive from their doctor or nurse. We refer to this as context. Context can be the things that patients think their healthcare providers need to be aware of. We’ll reflect on this context at the end as well but feel free to draw on these points when it makes sense for you. Please know that any topic is welcome for discussion. |  |
| Perceptions of compassionate care | For the purpose of this study, we define compassionate care as:  Awareness of another’s experience or need  Appraising one’s own role and abilities in an interaction  Being aware of context  but we want to hear in your own words, what does compassionate care mean to you? | How do you know you’re receiving compassionate care? /what does that feel like to you ? |
|  | To what degree has your family doctor/nurse expressed awareness of another’s (your) experience or need?    Can you walk me through what the interaction was like? | What made you feel that compassionate care was present? |
|  | To what degree has your family doctor/nurse considered their own role (responsibilities) and abilities in an interaction (with you)?  Can you walk me through what the interaction was like? | What made you feel that compassionate care was present |
|  | Primary care is unique in that you have the opportunity to interact with your family doctor/or nurse over time, how long have you known your family doctor/nurse? Can you describe the nature of your relationship with them? Do you have a strong relationship with them? | **Does** that impact your experience of compassionate care?  **Do** you think not having a strong relationship would impact your experience of compassionate care? |
|  | What aspects of health matter to you? |  |
|  | Please complete this sentence for me:  1. As a patient my responsibility is: ________________.  2. My physicians responsibility is: _________________. | How does your perception/experiences of compassionate care align with your perception of the responsibility of your family doctor/nurse? |
| **DIGITAL DIVIDE**  (trying to understand which level of the digital divide applies: access, capability, outcome) | How comfortable do you feel using technology? Would you feel adequately prepared (i.e. technical skill, access) to use technology when accessing care? | Is there anything else that could be done to make you feel more comfortable with technology? |
|  | What resources and conditions do you need to meaningfully engage in digital health? *Provide examples if needed i.e.,* video conferencing, high-speed internet | Do you have what you need to access care digitally? |
|  | Has your family doctor/nurse ever offered you digital health care visits? | How could using technology with your family doctor/nurse change your experience of receiving healthcare? |
| **MISTRUST/FEAR/STIGMA**  (trying to understand how their previous experiences affects their concerns about digital health) | Is there a reason as to why you have not engaged in digital healthcare so far? If there is, can you please share why that is? | What concerns do you have about using digital health? |
|  | How do you think your past experiences with compassionate care and your family doctor/nurse affect your willingness to use digital health technology? |  |
|  | In what instances do you think you would prefer to meet your family doctor/nurse in person versus virtually? | And how does that link back to having a compassionate interaction? |
| **CULTURAL COMPETENCE**  (how to tailor the digital health experience to the patient and ensure it is meaningful) | Under what circumstances would you be willing to use digital health? | What do you think needs to be done to make you feel comfortable enough to meaningfully engage in digital care? |
|  | How would you describe your ideal form of virtual care? What does that look like to you? | What format of digital health would be the easiest/most convenient for you to use? |
|  | Keeping in mind the role of context in compassionate care, how do you feel your personal background and lived experiences influences your experience of receiving compassionate care? We invite you to reflect on how your race, gender and other sociodemographic factors including language, and disability may impact both the care that you receive and your experiences within the healthcare system |  |
|  | How do you think your family doctor/nurse’s identity influences your experience with the healthcare system? | How does your family doctor or nurse’s background (race, linguistic, gender etc.) influence your comfort level?  What can your provider do better to provide you care that considers your context? |
| **COMMUNITY SUPPORT**  (what additional support is needed and if patients are open to it) | What support do you need to ensure you can engage in digital health care? |  |
| Closing | Is there anything else you want to share with me about compassionate care and digital health? |  |

Those are all the questions I had. Is there anything else you would like to add on this topic that we have not already covered?

Thank you for participating.

**Supplementary file 2: EMPaCT feedback report**

Community Consultation Report Feedback

OVERVIEW

This report outlines our reflections and changes undertaken as a result of the EMPaCT community consultations initiated by impact partners, Dr. Laura Desveaux and Kelly Wu. These consultations took place during May and June 2021, with the final consultation report provided to Dr. Desveaux and Kelly Wu in July 2021. The purpose of the consultations was to engage EMPaCT to inform the design and conduct of the research study titled: *Understanding the emerging intersection between digitally enabled and compassionate care*. The objectives of the consultation were to:

1. Advise on participant recruitment strategies, particularly on channels to recruit participants from underserved patient communities.
2. Provide feedback on the inclusivity and acceptability of participant recruitment materials.
3. Make recommendations on the interview guide questions.

REVIEW OF RECOMMENDATIONS AND PARTNER RESPONSE

| **EMPaCT**  **Recommendation** | **Response by impact partners** |
| --- | --- |
| To use informal and conversational language, specifically in patient-facing materials, to set the study context and in the interview guide questions | Language used in patient-facing materials such as recruitment emails, project information letter, and interview guides were reviewed against a readability index ([https://www.webfx.com/tools/read-able/)](https://www.webfx.com/tools/read-able/) to ensure they met a grade 8 language level. Taking into consideration the importance of context setting and using lay language in a manner that avoids otherizing participants, we made the preamble (*please see topic: Context setting in Appendix 2*) much shorter to help set the tone of the conversation as a welcoming and informal discussion between equals. We have maintained discussion points that probe around social determinants of health as we know these make a difference in the experience of care and we are hoping our study  can help add to the understanding of how they contribute. |
| To be responsive to different community- needs through appropriate, adaptive, and agile outreach | We have adopted EMPaCT’s suggestion to build relationships with community organizations (Appendix B of the report) as they have close connections to their patient population and are in the best position to provide appropriate and adaptive outreach on our behalf (if they wish to support the study). Our team remains available to support these organizations in reaching their patient population should they wish to engage us.  Recruitment posters for phase 2 of the study were described as “too busy”, with the research topic being confusing. We followed EMPaCT’s suggestions and removed unnecessary text and the term “compassionate care” which was flagged as confusing (*see Appendix 1*). Additionally, the poster adopts a broader framing so that patients don’t exclude themselves due to unclear eligibility (“Unsure about meeting your family doctor or nurse over a video call?”). The honoraria amount is also clearly  stated. |
| To provide options to participants, including choice of language, interview method, and gift card selection | Our team is committed to recruiting diverse participants and to reach out to patient participants who may not otherwise be accessible to research (recognizing this is a skill we will need to build over time). Participants who are not digitally enabled are unlikely to participate in a virtual interview so we have included a telephone number for study contact and will offer the option to conduct telephone interviews. Although we recognize the need to collect perspectives from patient populations who speak in other languages, it was not feasible for us to adapt in this way due to limited funding and the fact that we were halfway through data collection at the time of the consultation. There will be flexibility provided to participants for gift card selection  (i.e., range of store options). |
| To consider proxy questions to compassionate care as this may be understood differently by different  groups of people | This is something our team has struggled with and we are grateful for the feedback. We have included a definition of compassionate care at the beginning of the interview to give participants the opportunity to disagree with it or modify it. We have also modified interview questions to ask about the features of compassionate care (e.g., What aspects of health matter to you?) instead of using the term directly  (see Appendix 2). |
| To clearly state study scope and limitations so that gaps in research design can be illuminated for future funding and research | The scope of this study is to:   1. Understand whether and how digital care has shifted the patient-provider relationship. 2. Identify how and in what circumstances technology might amplify compassionate care. 3. Explore whether social identifies related to gender, race, or geography account for differences in experiences and perceptions.   We will interview patients who have had experiences with digital care as well as those who have not, to address these questions.  Due to COVID-19, interview options are limited to telephone or video call to all participants. This will be acknowledged as a limitation to data collection because it restricts recruitment and reduces access for participants, and we will highlight the need for future work to engage with participants in their communities using a  mechanism that is most accessible to them. |

NEXT STEPS

The updates described in this report were put into place and approved by the Women’s College Hospital Assessment Process for Quality Improvement Projects (APQIP) on August 3, 2021. We have reached out to 14 organizations for their interest in supporting participant recruitment. We have heard back from The Black Coalition for AIDS Prevention and Ontario Caregiver Organization. We have provided the REB approval letter, posters, a link to study recruitment Facebook post by Women’s College Hospital, and sample email text to support study circulation. We are beginning to analyze the data to understand what is common across participants and what is different. We anticipate completing all interviews for phase 1 by September 2021 and phase 2 by no later than October 2021. Engagement with EMPaCT so far has reached the “Involve” stage as illustrated in the Community Consultation Report - 5. Empact Engagement (adapted from the International Association of Public Participation (IAP2)). The research team is interested in working towards “Collaborate” and “Empower”, to create knowledge products and disseminate our findings to communities in an impactful and meaningful manner.

OPPORTUNITIES FOR FUTURE ENGAGEMENT

We would like to invite EMPaCT for the following opportunities for future engagement.

- A meeting in October where we can share our findings and EMPaCT members can:
  1. Share their reflections and interpretations of the findings so they can be further refined.
  2. Help us brainstorm how to create knowledge products that are most meaningful and helpful to patients and communities.

**Supplementary file 3: Recruitment list**

**Community organizations**

Women's College Hospital Family Practice; College of Physicians and Surgeons Ontario; College of Family Physicians of Canada (CFPC); Hope + Me (Mood Disorders Association of Ontario); Institute for Advancements in Mental Health (formerly Schizophrenia Society of Ontario); Canadian Mental Health Association; Ontario Caregiver Organization; Ontario Council of Agencies Serving Immigrants; Annishinaabe Health Toronto; The Anne Johnson Health Station; Black Health Alliance; Black Creek Community Health Centre; Black CAP; Taibu Community Health Centre; Queen West Central Toronto Community; Vibrant Healthcare Alliance; Access Alliance

**Ontario Health Teams (OHT)**

Algoma Ontario Health Team; All Nations Health Partners OHT; Brantford Brant OHT; Burlington OHT; Chatham-Kent OHT; Connected Care Halton OHT; Downtown East Toronto OHT; Durham OHT (Lakeridge Health); East Toronto OHT; EYRND OHT; Frontenac, Lennox and Addington OHT; Guelph and Area OHT; Hamilton OHT; Hills of Headwater Collaborative OHT; Kawarthas OHT; Mid-West Toronto OHT; Mississauga OHT; Muskoka and Area Ontario Health Team; Niagara OHT; North Toronto OHT

**Family Health Teams**

Akausivik Inuit Family Health Team; Algoma Nurse Practitioner Led Clinic; Algonquin Family Health Team; Amherstburg Family Health Team; Arnprior and District Family Health Team; Athens District Family Health Team; Atikokan Family Health Team; Aurora-Newmarket Family Health Team; Baawaating Family Health Team; Bancroft Community Family Health Team; Barrie and Community Family Health Team; Beamsville Medical Centre Family Health Team; Belleville Nurse Practitioner Led Clinic; Blue Sky Family Health Team Office; Bluewater Area Family Health Team; Bridgepoint Family Health Team; Brockton and Area Family Health Team; Burk's Falls Family Health Team; Burlington Family Health Team; Capreol Nurse Practitioner-Led Clinic; Care First Family Health Team; CarePoint Health; Caroline Family Health Team; Central Brampton Family Health Team; Central Hastings Family Health Team; Central Lambton Family Health Team; The Centre for Family Medicine; Chatham-Kent Family Health Team; City of Kawartha Lakes Family Health Team; City of Lakes Family Health Team; Clinton Family Health Team; Cochrane Family Health Team; Cottage Country Family Health Team; Couchiching Family Health Team; Credit Valley Family Health Team; Crosstown Family Health Team; Delhi Community Health Centre; Dilico Family Health Team; Don Mills Family Health Team; Dorval Medical Family Health Team; Dryden Area Family Health Team; Dufferin Area Family Health Team; Ear Falls Family Health Team; East Elgin Family Health Team; East End Family Health Team; East GTA Family Health Team; East Wellington Family Health Team; Elliot Lake Family Health Team; Emery Keelesdale Nurse Practitioner Led Clinic; Englehart and District Family Health Team; Espanola and Area Family Health Team; Essex County Nurse Practitioner Led Clinic; Etobicoke Medical Centre Family Health Team; Equipe de sante familiale academique Montfort; Eastern Ottawa Community Family Health Team; Family First Family Health Team; Fort Frances Family Health Team; Fort William Family Health Team; Four Counties Family Health Team; Georgian Nurse Practitioner Led Clinic; Georgian Bay Family Health Team; Georgina Nurse Practitioner Led Clinic; Grandview Medical Centre Family Health Team; Great Northern Family Health Team; Greenbelt Family Health Team; Greenstone Family Health Team; Guelph Family Health Team; Haileybury Family Health Team; Haldimand Family Health Team; Haliburton Highlands Family Health Team; Halton Hills Family Health Team; Hamilton Family Health Team; Hanover Family Health Team; Happy Valley Family Health Team; HarbourView Family Health; Harrow Health Centre Family Health Team; Health For All Family Health Team; Humber River Family Health Team; Huron Community Family Health Team; Huron Shores Family Health Team; Ingersoll Nurse Practitioner Led Clinic; Inner City Family Health Team; MICs Group of Health Services; Jane Finch Family Health Team; Kapuskasing and Area Family Health Team; Kawartha North Family Health Team; Kincardine Family Health Team; Kingston Family Health Team; Kirkland and District Family Health Team; Lakehead Nurse Practitioner Led Clinic; Lakelands Family Health Team; Lakeshore Community Nurse Practitioner Led Clinic; Lakeview Family Health Team; Leamington and Area Family Health Team; Leeds and Grenville Community Family Health Team; London Family Health Team; Lower Outaouais Family Health Team; Loyalist Family Health Team; Madawaska Valley Family Health Team; Maitland Valley Family Health Team; Mango Tree Family Health Team; Manitoulin Central Family Health Team; Manitouwadge Health Team; Maple Family Health Team; Marathon Family Health Team; Markham Family Health Team; Mattawa Family Health Team; McMaster Family Health Team; Minto-Mapleton Family Health Team; Mount Forest Family Health Team; Mount Sinai Hospital Family Health Team; Assiginack Family Health Team; New Vision Family Health Team; Niagara Medical Group Family Health Team; Niagara North Family Health Team; Nipigon District Family Health Team; Nord-Aski Family Health Team; North Channel Nurse Practitioner Led Clinic; North Durham Family Health Team; North Peel Family Health Team; North Perth Family Health Team; North Renfrew Family Health Team; North Shore Family Health Team; North Simcoe Family Health Team; North York Family Health Team; Northumberland Family Health Team; Oakmed Family Health Team; Ottawa Valley Family Health Team; Owen Sound Family Health Team; Parry Sound Family Health Team; Peninsula Family Health Team; Petawawa Centennial Family Health Centre; Peterborough Family Health Team; Peterborough 360 Degree Nurse Practitioner Led Clinic; Plantagenet Family Health Team; Points North Family Health Team; Portage Medical Family Health Team; Powassan and Area Family Health Team; Prescott Family Health Team; PrimaCare Family Health Team; Prince Edward Family Health Team; Queen Square Family Health Team; Queens Family Health Team; Rapids Family Health Team; Red Lake Family Health Team; Rideau Family Health Team; Sauble Family Health Team; Scarborough Academic Family Health Team; Sharbot Lake Family Health Team; Sherbourne Health Centre; Sioux Lookout Area Primary Care Team; Six Nations Family Health Team; Smithville Medical Centre Family Health Team; South Algonquin Family Health Team; South East Toronto Family Health Team; Southlake Academic Family Health Team; St. Michael’s Hospital’s Academic Family Health Team; St. Joseph's Urban Family Health Team; Star Family Health Team; Stratford Family Health Team; Summerville Family Health Team; Sunnybrook Academic Family Health Team; Sunset County Family Health Team; Superior Family Health Team; Taddle Creek Family Health Team; Temagami Family Health Team; Thames Valley Family Health Team; Thamesview Family Health Team; The Ottawa Hospital Academic Family Health Team; Westend FamilyCare Clinic Family Health Team; Tilbury District Family Health Team; Timmins Family Health Team; Toronto Western Family Health Team; Trent Hills Family Health Teams; Two Rivers Family Health Team; Upper Canada Family Health Team; Upper Grand Family Health Team; Village Family Health Team; Wawa Family Health Team; Welland McMaster Family Health Team; West Carleton Family Health Team; West Champlain Family Health Team; West Durham Family Health Team; West Nipissing Family Health Team; West Park Healthcare Centre; Windsor Family Health Team; Woodbine Family Health Team; Woodbridge Medical Centre Family Health Team
